# Comment on: Measuring the perceived duration of post-ejaculatory penile detumescence: a pilot study in a real-life setting

**DOI:** 10.1038/s41443-025-01159-7

**Published:** 2025-08-28

**Authors:** Mitsuru Komeya, Scott D. Lundy, Aaron W. Miller

**Affiliations:** 1 Department of Urology, Glickman Urological and Kidney Institute, Cleveland Clinic, Cleveland, OH USA; 2 Department of Cardiovascular and Metabolic Sciences, Cleveland Clinic, Cleveland, OH USA

**Keywords:** Reproductive signs and symptoms, Sexual dysfunction

The study by Suijker et al. [1] presents a timely and thought-provoking investigation into an often-overlooked component of male sexual function: the duration of post-ejaculatory penile detumescence, as perceived by men themselves. Using a participant-driven, real-life assessment strategy, the authors demonstrate that it is feasible to quantify this elusive physiological event outside of clinical or laboratory settings. Their findings not only provide preliminary normative values for detumescence duration but also introduce a patient-centered framework for understanding this phenomenon.

## Reframing erectile function through the lens of detumescence

Traditionally, men’s health research has emphasized the capacity to initiate and maintain erections, often measured by endpoints like erection hardness scores or intravaginal ejaculation latency time (IELT) [2]. However, the termination of an erection—its detumescence—is equally critical to understanding the complete sexual response cycle [3]. As the authors note, premature detumescence is a central concern for many men presenting with erectile dysfunction (ED), even when erection initiation is intact [4]. By bringing attention to the “fade-out” rather than the “rise” of the erection, Suijker et al. add nuance to the prevailing clinical discourse.

Notably, the conceptual framing of detumescence as a subjective, perceptual event—as opposed to a solely hemodynamic or neurochemical process—aligns with the broader shift in sexual medicine toward incorporating patient-reported outcomes into both diagnosis and treatment paradigms [5]. In this respect, the study echoes the shifts seen in the understanding of disorders like premature ejaculation, where subjective distress and perceived control now hold equal, if not greater, weight than stopwatch-based latency measurements [6].

## A methodological innovation with real-world validity

The Suijker et al. study’s design was simple: participants were instructed to self-measure, with a stopwatch, the duration between ejaculation and both the perceived onset and completion of penile flaccidity. Measurements were taken at the participant’s own residence and were free to choose how ejaculation was achieved. This method mirrors those used in large-scale IELT studies and appropriately trades some experimental control for real-world validity. Despite inherent limitations, the approach yielded fairly consistent results, with normal or near-normal distribution curves for both timepoints. Additionally, authors found a moderate correlation between the two detumescence phases (*r* = 0.47).

The investigators’ transparency in handling outliers and their conservative approach to data cleaning bolster the credibility of the findings. Furthermore, the use of validated measures (e.g., Erection Hardness Score) and the consideration of contextual variables (such as time of day, partner presence, and sexual satisfaction) enrich the dataset without overextending the scope of this pilot effort.

## Limitations as opportunities for future research

While the feasibility of such self-reported measurement is now established, several limitations inherent to this pilot design invite further inquiry. The sample size (*n* = 83) and recruitment via social media yield a predominantly young, tech-savvy, and presumably sexually active cohort (mean age 27 ± 9 years), potentially limiting generalizability. Future research would benefit from more stratified sampling, including older men and clinical populations such as those with ED or priapism. Since aging is a risk factor for ED, investigating the relationship between age and penile detumescence duration would be of particular interest.

Moreover, the subjective nature of the start and end points of detumescence introduces a potential for measurement ambiguity. Some participants noted confusion regarding when exactly to initiate or terminate timing, which may reflect the lack of discrete physiological cues marking these transitions. Nonetheless, this ambiguity is arguably intrinsic to the phenomenon under investigation. Much like sexual satisfaction or arousal, detumescence may defy precise delineation, thus necessitating subjective reporting [7]. Future studies may explore whether anchoring the definitions of “onset” and “complete” detumescence to specific tactile or visual sensations improves measurement reliability.

Importantly, the present study did not collect detailed psychosexual or medical history, including lack of physical activity, diabetes, hypertension, dyslipidemia, smoking, or chronic alcohol consumption, nor did it assess potential correlates such as testosterone levels, medication use, relationship status, or psychological distress. These factors are well-documented influences on erectile function [8] and should be incorporated into follow-up studies aiming to move beyond feasibility and toward causal inference.

## Bridging the gap between perception and physiology

Perhaps the most intriguing implication of this study is the potential to bridge subjective sexual experiences with objective physiological mechanisms. Prior research has described detumescence in terms of cavernous blood outflow and sympathetic tone [3]. Suijker et al. open the door to examining how these processes are experienced—and timed—by individuals in real-world settings. The reported durations (mean 58 s to onset; 181 s to complete flaccidity) suggest a multi-phase process, paralleling findings from animal studies that describe three distinct phases of detumescence [9]. Identifying which phase is impaired—based on the patient’s subjective perception—may allow for a more precise, mechanism-based diagnosis of ED. Future multimodal studies might combine real-time penile rigidity monitoring (e.g., RigiScan or Doppler ultrasound) with participant-reported perception to more accurately map subjective experience onto physiological change.

Further, comparing detumescence durations between ejaculated and non-ejaculated sexual activity could illuminate the role of orgasmic neurochemistry in maintaining post-coital tumescence. Previous research suggests that ejaculation may prolong detumescence [10], as mentioned by the study authors, and this hypothesis warrants systematic testing in both experimental and naturalistic settings.

## Clinical relevance and the road ahead

The clinical utility of measuring detumescence duration is not yet established, but several avenues merit exploration. First, changes in detumescence over time may serve as an early marker of endothelial or smooth muscle dysfunction, potentially prefiguring ED [11]. As detumescence duration is associated with the contractile function of penile arteries, it may also reflect broader vascular health, suggesting a possible link with systemic vascular diseases that warrants further investigation. Second, unusually long detumescence may indicate neurovascular or hormonal imbalance and may aid in differentiating subtypes of priapism [12]. Third, as patient-centered outcomes grow in importance, perceived detumescence duration could become a meaningful endpoint in trials of ED therapies.

While the present study focused solely on men, the relationship between detumescence duration and sexual satisfaction is a compelling topic for future research. There may also be potential to extend this concept to female physiology, particularly in studying clitoral tumescence and detumescence. Such an approach could provide valuable insights into female sexual health and satisfaction.

Ultimately, this pilot study lays the groundwork for a more nuanced, real-world, and patient-aligned understanding of male sexual physiology. As sexual medicine continues to evolve, efforts like this—which prioritize the patient’s lived experience and harness simple yet effective tools—will be instrumental in closing the gap between clinical observation and subjective reality.
